# The Human Cerebellum as a Hub of the Predictive Brain

**DOI:** 10.3390/brainsci11111492

**Published:** 2021-11-12

**Authors:** Daniele Gatti, Luca Rinaldi, Laura Ferreri, Tomaso Vecchi

**Affiliations:** 1 Department of Brain and Behavioral Sciences, University of Pavia, 27100 Pavia, Italy; luca.rinaldi@unipv.it (L.R.); vecchi@unipv.it (T.V.); 2 Cognitive Psychology Unit, IRCCS Mondino Foundation, 27100 Pavia, Italy; 3 Laboratoire d’Étude des Mécanismes Cognitifs, Université Lumière Lyon 2, 69767 Lyon, France; laura.ferrer@univ-lyon2.fr

**Keywords:** cerebellum, prediction, cognition, language, music

## Abstract

Although the cerebellum has long been believed to be involved uniquely in sensorimotor processes, recent research works pointed to its participation in a wide range of cognitive predictive functions. Here, we review the available evidence supporting a generalized role of the cerebellum in predictive computation. We then discuss the anatomo-physiological properties that make the cerebellum the ideal hub of the predictive brain. We further argue that cerebellar involvement in cognition may follow a continuous gradient, with higher cerebellar activity occurring for tasks relying more on predictive processes, and outline the empirical scenarios to probe this hypothesis.

## 1. Introduction

Predicting what is going to happen in the future in order to optimize neurocognitive resources for upcoming tasks is perhaps one of the most important functions that the human brain has to deal with in order to successfully adapt to the surrounding environment [1,2]. Accordingly, the interest in predictive processing dates back to the 19th century, with William James’ definition of sensory anticipation as *preperception* and his postulation that certain brain areas could be pre-activated in order to optimize resources, thus reducing the subsequent processing load [3]. Prediction can be defined as a process that incorporates information about the past or the present, and that generates the relevant information for coping with future (environmental, bodily or mental) states [4]. Consistent with this, experimental evidence is available for predictive involvement in almost every human function (e.g., for motor control: [5]; for attention: [6]; for language: [7]; for executive functions: [8]; for memory: [9]), demonstrating the pervasiveness of prediction in human cognition.

Notably, despite different areas of the human brain having been shown to participate in predictive mechanisms, the cerebellar circuit likely represents the primary candidate for coordinating such predictive computations (for a review: [10]). Indeed, despite almost the entire brain being to various degrees involved in predictive processing (i.e., with brain areas spanning from the frontal to prefrontal, temporal and parietal regions, together with other cerebral structures such as the hippocampus, the cerebellum, the basal ganglia, the insula and the amygdala; see [4]), here we propose that the cerebellum may be considered as the principal hub of the predictive brain [11].

While the cerebellum has indeed been traditionally associated with motor aspects of prediction, in the last decade, important evidence has emerged extending its predictive role to higher-order cognitive processes ([10,12]; and for a meta-analysis see [13]). This evidence corroborates previous theoretical accounts, according to which the cerebellum is able to “regulate the speed, capacity, consistency, and appropriateness of mental or cognitive processes” ([14], p. 1183). Accordingly, the cerebellum is thought to perform the same basic processes across the various motor and non-motor functions ([12]; but for a divergent view, see [15]), a key feature making this region as the ideal orchestra director of the predictive brain.

In the following sections, we will thus first briefly review the available evidence supporting a generalized role of the cerebellum in prediction. We will then discuss how cerebellar anatomo-physiological as well as functional properties can account for its involvement in predictive computations for both motor and nonmotor domains. Next, we will move on by maintaining that cerebellar involvement in cognition follows a continuous gradient, with higher cerebellar activity occurring for those cognitive tasks relying more on predictive computation. We will ground this proposal in recent evidence indicating that cerebellar activation increases with enhanced task demands relying on predictive mechanisms. Finally, we will also discuss how our proposal makes testable predictions for subsequent empirical research.

## 2. Cerebellar Prediction across Motor and Non-Motor Domains

Currently, our knowledge about the predictive computations performed by the human cerebellum is still mainly based on motor functions. Several studies have shown that patients with lesions over the anterior parts of the cerebellum exhibit disturbances in motor accuracy and coordination, such as disorders of saccades control, speech production (e.g., anarthria), limb movements, posture and gait (for a review see: [16]). For example, cerebellar patients typically exhibit hypermetria or hypometria, impaired timing of movements, overall slowness, and increased curvature of trajectories [17]. These findings have led to the hypothesis that the cerebellum contributes to timing processing, sensory acquisition and motor coordination across motor functions. In particular, the cerebellum is thought to be involved in the internal representation of the consequences of an action, that is, comparing what was predicted and what actually occurred (i.e., with the generation of potential signal errors in case of a mismatch between the two). The cerebellum would thus make motor coordination possible, in that it would use the efferent copy (i.e., an internal copy of the motor command) to simulate the sensory consequences of the movement and would then compare this copy with the actual feedback generated [18]. For instance, in grasping tasks, patients with cerebellar lesions show impaired performance in controlling the amount of force used when grasping an object [19,20], likely reflecting impaired anticipation of the consequences of actions and synchronization of the musculoskeletal system during voluntary movements [21]. Recently, Tanaka and colleagues ([22]; for evidence on deterioration of predictive motor control in ataxic patients, see [23]), provided direct evidence for the contribution of the cerebro-cerebellum to predictive processes. In particular, this study demonstrates that the current firing rates of dentate cells from the cerebellum can predict the future firing rates of mossy fibers, hence supporting the forward-model hypothesis [10].

Notably, several basic functions have been linked to cerebellar involvement also across non-motor processes, including the generation of internal models, as well as the processing of sequential and temporal information (for review and experimental evidence, see [24,25,26,27,28]). In particular, the past few years have seen a rapid increase in the number of studies exploring cerebellar involvement in predictive computation in non-motor, cognitive domains. Strong evidence for cerebellar involvement in predictive cognitive processing comes from studies focusing on the language domain. The ability to create and update internal models may indeed be crucial for error monitoring and fluency during speech production. Accordingly, in a seminal study, Lesage and colleagues [29] combined eye-tracking and transcranial magnetic stimulation (TMS) and showed that TMS over the right cerebellum caused a reduction in the anticipatory advantage for predictive sentences as compared to control stimulation. More specifically, cerebellar TMS impaired predictive performance for predictable sentences (e.g., “the boy will eat the cake”) but not for unpredictable sentences (e.g., “the boy will move the cake”). These results were recently replicated using transcranial direct current stimulation (tDCS) [30]. Similarly, Moberget and colleagues [31] found that cerebellar activation significantly increased following a violation of linguistic contextual predictions (e.g., “two–plus–two–is—APPLE”) compared with contextually correct predictions (e.g., “two–plus–two–is—FOUR”). This increased activation can be interpreted as the effort made in matching the expected event with the one actually occurring and, hence, in the extent to which the task at hand was relying on predictive computations. Finally, cerebellar beta-frequency TMS increases N400 correlates of semantic prediction, thus suggesting enhanced discrimination of semantic context predictability [32].

Further evidence comes from studies investigating verbal working memory [33] or attentive processing [34]. For instance, in a recent study by Sheu and colleagues [33], participants were asked to memorize an array of six letters and then, after a short delay, to report the letters in the correct position. The results show that cerebellar TMS impaired participants’ performance, resulting in prediction errors in the phonological loop. In another recent study, Mannarelli and colleagues [34] showed that after cathodal cerebellar tDCS, the efficiency of the executive network is reduced and the ability to process complex stimuli—in which conflict signals or errors are present—is impaired. Together, these findings indicate that the cerebellum is involved in predictive error processing, as well as in the coordination of the areas involved in the perception of conflicting signals.

Preliminary neuroimaging evidence suggests a possible role of the cerebellum in predictive functions in social cognition, as the violation of social norms (i.e., a process that requires to compute whether the action could be construed as a violation and, crucially, whether the action was intended) has been shown to elicit fronto-temporo-cerebellar activations ([35]; see also [36,37]). In line with this preliminary evidence, cerebellar tDCS has been demonstrated to modulate participants’ performance in predicting actions embedded in highly informative contexts [38]. Future studies are nevertheless still needed to probe cerebellar involvement in predictive processing across different aspects of social cognition, as well as whether cerebellar involvement in cognitive functions is based on the very same predictive computations operating for the motor domain (for preliminary evidence, see [39]).

## 3. Anatomo-Physiological and Functional Features Make the Cerebellum the Ideal Predictive Hub

Why would the cerebellum represent the ideal hub for the predictive brain? In this section, we propose that specific cerebellar anatomo-physiological and functional features make this part of the brain the ideal candidate for coordinating predictive computations for the whole brain.

First, compared with the 21–26 billion neurons in the cerebral cortex, the impressive cerebellar neural machine has around 100 billion neurons [40], thus making this relatively small part of the brain an ideal hub for demanding tasks relying on predictive computation. Second, the composition of cerebellar cortex is markedly different compared with the cerebral cortex. The cerebellar cortex has a homogeneous and uniform microstructure [41]—that is, contrary to the cerebral cortex, the various lobules composing the cerebellum do not differ in terms of microstructure. Based on this, it has been proposed that the cerebellum performs the very same computational processes across all the domains in which it is involved ([12,14]; for a review, see also [10]). Such a view has been also supported by recent lesion studies indicating that cognitive and affective symptoms that arise after cerebellar dysfunctions follow a similar pattern of abnormality as for motor symptoms [39]. This is compatible with the proposal that the cerebellum performs similar predictive computations across different cognitive and motor domains.

Another key feature of the cerebellum, partially deriving from such a structural and functional uniformity, is the segregation of the lobules composing the cerebellum through their extensive connections with the cerebral cortex (i.e., cerebellar regions connected to motor areas are involved in motor processes, while cerebellar regions connected to cognitive areas are involved in cognitive processes; [12]). Several studies have indeed supported a functional double dissociation between the anterior and posterior cerebellar lobes, with the former connected to motor areas and the latter to non-motor areas (for a review, see: [42]). In addition to this, it was also recently demonstrated that cerebro-cerebellar connections are segregated [43,44,45], thus allowing specific cerebellar areas to participate in specific functions. Accordingly, verbal and semantic processing are generally right-lateralized in the cerebellum ([46,47]; for evidence involving verbal and spatial working memory, see also [48,49]), whereas, in the cerebral cortex, they are left-lateralized [50,51], reflecting crossed cerebro-cerebellar connections [52,53]. Furthermore, while lesions in anterior cerebellar areas result in clear motor impairments mostly linked to movement coordination, lesions in posterior cerebellar areas typically result in a set of symptoms involving deficits across executive functions, language, attention, social cognition and spatial processing (this syndrome is generally referred to as cerebellar cognitive-affective syndrome (CCAS), or Schmahmann’s syndrome [54]; e.g., for causal evidence of cerebellar involvement in these processes, see [55,56,57]) that would not be simply explained by concurrent motor impairments [58]. Together, this evidence indicates the specific cerebellar involvement in different cognitive functions, likely because of the segregated connections with the various brain areas supporting such functions. This is a potentially paramount feature for an hub that coordinates predictive processes for different cognitive functions.

Finally, on the functional side, the cerebellum can be considered as a multimodal structure. Several studies have shown that the cerebellum is involved in the integration of proprioceptive, vestibular, visual and motor efference information in order to create a unified, multimodal representation of a target event [59]. More importantly, cerebellar involvement has been reported in multisensory integration [60], sensory–motor integration [61], as well as semantic integrative processes [62]. Such an extensive participation in integrative processes may demonstrate the fact that the cerebellum acts as a central coordinator of the entire predictive system, as this structure has been shown to be able to store and use flexible representations of objects in ecological contexts [63].

Together, the evidence presented here supports the proposed view of the cerebellum as a central hub of the predictive brain. In the next section, we will review recent experimental evidence and make our proposal more specific by maintaining that cerebellar involvement follows a continuous gradient depending on the specific predictive demands required by the task at hand.

## 4. A Gradient of Cerebellar Involvement

Despite the growing number of studies that support cerebellar involvement in predictive processes across motor and non-motor domains [12], the specific mechanisms of such an involvement across the various domains, as well as its possible interplay with the rest of the nervous system, are not yet clear. The role of the cerebellum in predictive processes has indeed been largely supported by studies comparing experimental conditions clearly relying on predictive computations with those in which predictive computations were not required and, thus, on an all-or-nothing basis. In this section, we rather propose that that cerebellar involvement in cognition follows a continuous gradient, with higher cerebellar activity occurring for those cognitive functions or experimental tasks relying more on predictive computations.

In grounding this proposal, we first note that cerebellar activation increases with increasing task demands. In a seminal functional magnetic resonance imaging (fMRI) study, Xiang and colleagues [64], using three different discrimination tasks assessing semantic discrimination with different levels of difficulty, reported stronger cerebellar activation (i.e., in terms of both activation volume and signal intensity) in more difficult tasks. These findings were recently extended in another fMRI work [65] by showing that cerebellar activation increases with higher central executive demands in verbal and non-verbal working memory tasks (e.g., n-back tasks). In addition, using the same paradigm adopted by Moberget and colleagues [31], another recent study reported that tDCS modulates BOLD activation patterns only during predictable sentences compared with non-predictable ones [66]. That is, compared with control stimulation, anodal stimulation increased right cerebellar activation during semantic prediction and supported a central role for the cerebellum in predictive cognition, enhancing resting-state functional connectivity between hubs of the language network.

Other evidence comes from a recent study [67] employing TMS over the right cerebellum during the recognition phase of the Deese–Roediger–McDermott paradigm (DRM [68]), a typical false memory task in which participants are asked to memorize several lists of words and then to perform a recognition task. The words that compose each list are associatively related to a non-shown word (called critical lures), which is generally falsely recognized [69,70]. In two experiments, Gatti and colleagues [67] found that cerebellar TMS selectively affected participants’ discriminability for critical lures (i.e., the false memory items) without affecting participants’ discriminability for unrelated words. More critically, the higher the semantic association between new and studied words (i.e., how accurately new words could be predicted on the basis of the semantic content of studied words), the stronger the effect of the cerebellar stimulation [67].

Notably, the concept of a gradient of involvement can take multiple forms, and has been recently employed to account for the functional organization of the cerebellum. For instance, recent evidence provided support for the existence of a gradual functional organization of the cerebellum that spans from unimodal (e.g., motor) to trans-modal areas in a sensorimotor-fugal manner [71]. Similarly, a functionally relevant gradient within the posterolateral cerebellum has been documented, with motor-adjacent cerebellar regions associated with the control of current actions and motor-distal cerebellar regions associated with the preparation for future goals and actions [72]. Here, we predict that the cerebellum is gradually involved in cognitive tasks depending on the extent to which these tasks rely on predictive processing.

Taken together, these findings suggest that cerebellar involvement in predictive processes may follow a continuous gradient, with tasks heavily relying on predictive processing eliciting a higher cerebellar activation. This proposal may explain the relatively high heterogeneity of motor and non-motor functions ascribed to cerebellar activity (i.e., if the task relies on predictive processes, the cerebellum would presumably be involved), as well as its degree (the stronger the predictive component, the higher the cerebellar involvement).

## 5. Future Directions and Challenges

To empirically test this proposal, future studies may develop ad hoc tasks that differently manipulate the predictive load required. This could be ideally investigated in two cognitive domains that strongly rely on predictive operations and that have been recently ascribed to cerebellar functioning, such as language and music domains [73,74]. Language and music indeed share many commonalities, in that their repetitive architecture makes it possible to easily detect predictive components. As a consequence, in these domains, one may operationalize the predictiveness of relevant cues by computing indexes such as word-frequency, typical word co-occurrence or pitch co-occurrence. These indexes can, in turn, provide a continuous measure quantifying how much expected predictions are violated or confirmed. For instance, the use of distributional semantic models from computational linguistics may represent an ideal tool for quantifying the level of predictiveness in the language domain [75]. The architecture of these models is indeed grounded in associative learning mechanisms, which are in turn crucial for cerebellar internal models [73].

**Figure 1:**
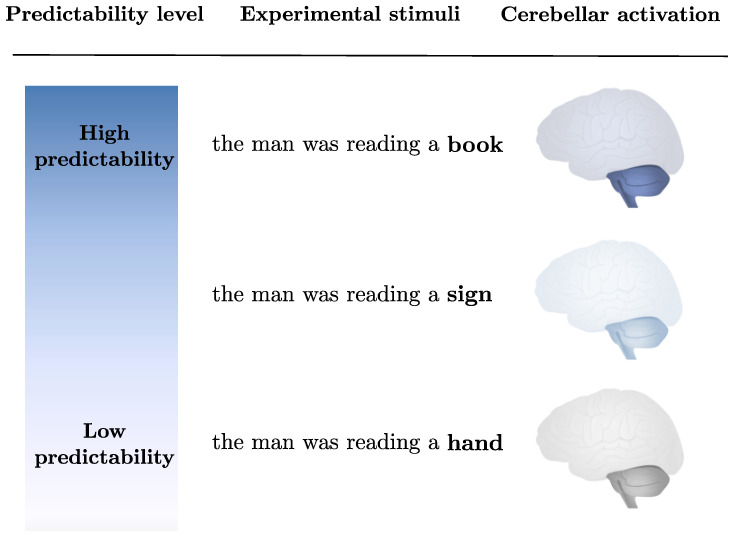
Schematic representation of the alleged gradient of cerebellar activation as a function of the language-based predictability. In particular, for the language domain, we expect cerebellar activation to be related to the predictability of the sentence, with higher activation for more predictable words.

Within the language domain, it would be relatively easy to build different linguistic stimuli relying on predictive components to various degrees and then investigate the relative cerebellar involvement. For example, one may use fMRI to explore cerebellar activation in sets of sentences with a different gradient of predictability ranging from low (e.g., “the man was reading a *hand*”) to high (e.g., “the man was reading a *book*”) (see: Figure 1 for a schematic representation). These future studies may also manipulate the cognitive level of prediction (i.e., surprisal vs. semantic similarity vs. compositionality, e.g., [76,77]), thus directly investigating the mechanisms underlying cerebellar activity.

Similarly to language, music is a complex stimulus that occurs over time and offers a great opportunity to understand predictive processes [78,79,80]. While listening to music, we constantly generate hypotheses about what could happen next in terms of both temporal (e.g., rhythms) and melodic (e.g., notes or chords) events [81]. The cerebellum, likely because of its ability to detect changes and deviations in sequential events [27,82], has been shown to play a crucial role in rhythmic perceptual and motor entrainment (i.e., the interaction of the external rhythm of the music with an internal body rhythm of the listener; see, e.g., [83], which is pivotal for the generation of musical expectancies [84,85,86]). Interestingly, musical regularities can be manipulated to different degrees, for example through variations in the metric complexity [87], or via violations of pitch expectations [88]. This type of stimuli, varying in predictive load, can be easily adapted to an fMRI paradigm in order to explore the cerebellar involvement in predictive processes. In a continuous music listening task, participants are asked to listen to less, moderately, and highly predictable rhythms. Higher cerebellar activation should be observed for highly predictable as compared to moderately predictable stimuli, and for moderately predictable as compared to less predictable stimuli. As musical training has been shown to lead to a predictive listening advantage, also associated with changes in cerebellar connectivity [89,90], the gradient of cerebellar involvement could be further explored by investigating the differences between musicians and non-musicians. In particular, the expected effect (i.e., higher cerebellar activations for highly versus moderately versus less predictable excerpts) should be stronger in a group of trained musicians as compared with non-musician participants.

As stated above, language and music can be considered as two ideal domains to test our hypotheses, since they both allow for a formal, quantitative definition of the associative and predictive relationship between stimuli. However, cerebellar involvement has also been reported across several other domains, as in the case of the three main components of executive functions (e.g., inhibition, multitasking and working memory; [42,91]). Accordingly, the gradient hypothesis outlined here could also be tested on executive functions, possibly employing predictive tasks already established in the cerebellar literature [33].

## 6. Conclusions

Prediction is involved in almost every human function, from more basic ones, such as in the case of motor coordination, to more complex ones, such as in the case of semantic memory. Here, we argue that, despite several studies reporting evidence for the involvement of a large number of brain areas in predictive processes, the cerebellum should be considered as the principal hub of the predictive brain. This proposal was grounded in the uniformity of cerebellar cortex microstructure and its segregated connections with the cerebral cortex, as well as in its functional role, which makes the cerebellum the ideal director coordinating predictive computations. Moreover, to explain the relatively high heterogeneity found in cerebellar activation across various motor and non-motor tasks, we proposed that cerebellar involvement in predictive processes follows a continuous gradient, with specific predictions that were outlined to fully probe this possibility in the language and music domains. This is especially desirable to ultimately improve our understanding of a brain system such as the human cerebellum, which has been traditionally associated with motor aspects but whose participation in cognitive processes is arousing more and more interest.
